# Entero-Colitis of Infancy*Read before the Georgia Medical Association at Macon, April 21, 1897.

**Published:** 1897-06

**Authors:** M. A. Clark

**Affiliations:** Macon, Ga.


### ENTERO-COLITIS OF INFANCY.*

BY M. A. CLARK, A.M., M.D.,
MACON, GA.

When we consider how many little lives are sacrificed by this dread disease, we are impressed that it cannot be too often discussed. Hence if I do no more than emphasize what has already been taught with regard to it, I shall not have imposed upon the time of this Association. It is my purpose to give you my views as the result of a careful study of numerous cases. I shall devote the most of my time to the treatment, it being the most important part of the disease.

I prefer the term entero-colitis to intestinal catarrh, because it more fully conveys what the pathology of the disease is. While in adults and children we recognize enteritis and colitis as separate and distinct diseases, yet in infants the symptoms are not sufficiently well marked to enable us to differentiate them. In fact, as J. Lewis Smith says: "In the majority of instances there exists an inflammation of both the small and large intestines, the one rarely existing without the other."

This inflammation may be ulcerative, membranous, or, as is more frequently the case, a simple inflammation of the mucous and submucous linings of the intestines.

*Causation.*—Cold is often a cause of entero-colitis, and, in a majority of the severer cases, it plays an important part. The

*Read before the Georgia Medical Association at Macon, April 21, 1897.

most important factors, however, in the causation of this disease are temperature and diet.

The summer heat, without doubt, influences very markedly the cause of this disease, as it invariably occurs in its intensity during the summer months. It is most fatal during July and August, and abates as cooler weather comes on. It is, however, not so much the heat itself as the effect it has upon the atmosphere and diet.

After all, it is the diet that is the most important cause. This is proven by the fact that the majority of fatal cases are bottle-fed infants. Of 1,943 fatal cases reported by Holt, only three per cent. were wholly breast-fed. All writers agree that outside of the cities, especially in the rural districts, it is of far less severity. Some seem to think it of little importance in these districts. We will all readily agree that it is far more fatal in cities, but we must insist that its gravity is such as to cause us deep concern in our towns and villages. In this fast age of ours so many mothers are unable to nurse their babes that artificial feeding must be relied upon. Consequently entero-colitis is now a disease that demands careful thought and attention even by the country practitioner.

Age is a predisposing cause. The greatest number of cases occur between six and eighteen months. It is less frequent up to the third year, after which it is rare.

Teething is usually given as a predisposing cause. Some authors even go so far as to explain that the excess of salts in the saliva produces a diarrhea that is beneficial to the patient. How often it has been said that the baby is sick with teething. Even the physician, in answer to the question of the anxious mother as to what is the matter with the baby, replies, "Teething." It is proverbial with the laity that the second summer is very trying with infants because they are cutting teeth. I wish to say most emphatically, teething is wholly a physiological process, and has nothing to do with the cause of this disease.

During the process of dentition nature is preparing the alimentary canal for the important functions of digestion, and, because of this evolutionary process, the whole mucous membrane is susceptible to the slightest irritant. The teeth have absolutely nothing to do with this process, and, if kept clean and sound, have nothing to do with the bowel troubles of infancy. Let us use every available opportunity to eradicate such ideas from the minds of the laity. Many little lives have been allowed to languish and die because their mothers thought they had to be sick while teething.

According to Booker and Vaughan, bacteria are found in the stools. Hence there is no doubt that bacteria play an important part in the cause of the bowel diseases of infancy.

*Symptoms.*—Onset is usually gradual—languor, fretfulness, and slight rise of temperature. The diarrhea is so mild at first that it is considered of no importance, so thoroughly prevalent is that erroneous idea that the bowels must be loose during dentition. Let us impress upon all mothers, that any diarrhea, however slight, during teething, is a symptom of disease, and should have the attention of the physician. By thus acting we will save ourselves many anxious moments and relieve many little sufferers before it is too late.

The stools are variable at first—yellow, then brown, then green, or they may be green from the onset. At first there may be considerable fecal matter, then stools become small, with mucus and blood, sometimes pus and shreds of membrane. The blood is usually from congestion of blood-vessels and straining. In mild cases, from five to six stools in twenty-four hours; in severe cases, from twenty to thirty.

If inflammation extend to the rectum, there is pain and burning before and after stools.

Temperature is usually slight, but frequently runs up to 102° or 103° F. After a prolonged attack it may be subnormal.

The tongue is first moist and covered by a brownish or grayish fur, then becomes dry, and in severe cases red and swollen.

Vomiting is not apt to occur till after the first week unless the stomach is involved. Persistent vomiting is usually a grave symptom. The skin is dry and cool except over the bowels, where it is hot. It is always cool in the stage of collapse. Thirst is usually very marked, especially where stools are very frequent and fever high. Pulse varies with the intensity of the disease. Boils and erythema are frequent complications. Hypostatic congestion of the lungs with cough may result from prolonged recumbency and enfeebled heart's action.

Spurious hydrocephalus is ushered in with vomiting, rolling of eyes, tossing of head, mouth open, eager to drink anything offered, even nauseous doses, drowsiness, stupor, pulse frequent and feeble, usually followed by death.

If case is mild, recovery results in two or three weeks. Severe cases may run on five or six weeks as subacute or chronic.

*Diagnosis.*—By careful attention to the symptoms and history of the case, diagnosis is usually easy.

*Prognosis.*—Mild cases tend to recovery. When complicated by bronchitis, hypostatic pneumonia, or gastritis, the prognosis is grave. Spurious hydrocephalus is always very grave.

*Treatment.*—The first and most important duty of the physician is to prevent disease. So in this disease as well as all others we must first consider preventive treatment.

*Clothing.*—I wish to emphasize this proposition: Every infant should wear throughout all seasons flannel next to the body till the period of infancy is past and childhood well established. Indeed, I would hardly go wrong to say that every human being should wear flannel next to the body every day and all day. It is hardly necessary for me to call your attention to the protection given by wearing flannel next to the body. Do not be content with the usual flannel skirt, but see that there is a close-fitting flannel garment extending over the chest and whole abdomen.

It is no infrequent thing to see lying on the floor kicking up its heels, a little babe with no clothing on the bowels save a thin cotton dress. As a result, the bowels become chilled and thus often begins a troublesome bowel disease. While this may not of itself cause a serious entero-colitis, yet it so affects the bowels that the slightest irritant precipitates the grave disease. Insist too that the flannel must be worn at night, this being more necessary than in the day.

This may seem to be very unnecessary instruction. Allow me to remark that the careful observance of little things will often enable us to prevent the graver symptoms of disease. In the treatment of every infant instruct the mother as if she knows nothing of the management of infants. Away with the false idea that old women know more about managing babes than the doctors know.

*Diet.*—The all important factor, not only in the cause of this disease, but also in the cure of it after it is produced, is diet. In the majority of cases the infant is bottle-fed, yet we find some severe cases that are breast-fed. In every case we must examine the milk to see that it is suitable, and when not have the diet changed at once. In those dependent upon artificial feeding, the finding of a suitable food is no small undertaking. All are agreed that next to mother's milk, cow's milk is the best food for infancy.

The many ways of preparing the milk have been so well shown by Smith, Rotch, Holt and others, that I need only refer you to their valuable works. Yet we must bear in mind that each case is an indvidual one and must be treated as such. No fixed rules can be formulated. The physician must be attentive and studious and must use the very best judgment and skill.

In the large cities where the scientifically prepared laboratory foods are available, the problem of diet is much more easily solved, and the benefit to the sick is indeed great. With us it is different. We must instruct the mothers how to prepare the diet and must depend upon their following our instruction.

When I know the milk has not been exposed to foul air, I prefer pasteurizing to sterilizing it because it is more palatable and more easily digested. In no case do I give the milk alone, but always diluted with some cereal, preferably barley-water. In infants under one year, I use equal parts of fresh sweet milk and barley-water; over one year, two or three of milk to one of barley-water. It should be given at regular intervals and in small quantities. This is very important.

In severe cases where the milk thus prepared does not agree with the patient, after adding the barley-water, predigest with extract of pancreas and sodium bicarbonate. If this is not assimilated, and especially if there is vomiting, give *no milk at all.* Then we must resort to some prepared food. Often a little barley or rice-water with the white of an egg and a little salt is well borne and is nutritious. I have had excellent results from the use of bovinine, 10 to 20 drops in a little water every two or three hours. Horlick's malted milk is a most excellent preparation, and when properly diluted and frozen, it is taken with eagerness by the little sufferer and is well assimilated. Milk prepared according to Rudisch's method, a half teaspoonful of dilute hydrochloric acid to a pint of water and a quart of milk, is often well taken.

In every case the physician must have at his command all available resources and must use his brains to enable him to decide what is best suited to that special case. As Jacobi has wisely remarked: "No fixed rules can supply the place of brains."

So much has been said by authors about the cleansing of the bottle and nipple that it is not necessary for me to more than mention it. Allow me to say that the physician who allows the babe to use the rubber tube attachment, is guilty not only of the grossest carelessness, but also of the death of his patient, for indeed the babe, who must take its food through this most certain source of infection, must needs die. Because of the ease with which it is kept clean, I advise the use of the bottle called "The Best."

Where the child is more than one year of age, I advise the feeding with a spoon because of the difficulty of getting the bottle and nipple well cleansed.

*Medicine.*—At the onset of the disease, when the tongue is heavily coated and the stools of a greenish color, a laxative is usually indicated. For a child under one year I prefer the following:

℞ Salol.................................................gr. x.
Hydrarg. cum creta.................................gr. xv.
Sacchari albi.........................................gr. xij.
M. et ft. chartulæ, xij.
Sig. One powder every two hours.

In this I get the laxative and antiseptic effects of the mercury and the antiseptic effects of the salol.

It is claimed by some that no good results from the use of salol. I am sure, however, that I have had decided results and no bad effects from its use during the first stage of the disease.

I then give to a child under one year the following:

℞ Tannigen.............................................gr. xx.
Pul. peptenzyme....................................gr. xxx.
Syr. aurantii..........................................ʒ iv.
Aquæ, q. s.............................................℥ ij.
M. Sig. One teaspoonful every three or four hours to control bowels.

I find tannigen a most valuable remedy for any diarrhea. It is insoluble in the stomach, and in the intestines is converted into tannic acid, the best astringent. By its use I have overcome what would have proved to be prolonged and severe attacks of this disease. The peptenzyme is an aid to digestion and tends to suppress nausea.

In severe cases I alternate with the following:

| | | |
|---|---|---|
| ℞ | Resorcini................................................ | gr. xx. |
| | Bismuthi subnitr............................................. | ʒ vj. |
| | Elix. panpeptic. ............................................. | ʒ j. |
| | Syr. aurantii, q. s............................................ | ℥ iij. |

M. Sig. One teaspoonful every three or four hours.

Bismuth is *par excellent* when there is much tympanites. With regard to bismuth, let me remark that we are often disappointed in its results because we give too small doses. I do not hesitate to give to a child of six months grains xx. of the subnitrate every two hours when the stools are frequent and the bowels are tympanitic. The salicylate is recommended by some as being more antiseptic. I prefer the subnitrate, as I get better results from its use.

When the tannigen is pushed, it is rarely necessary to resort to the bismuth. Give it till the bowels are checked, and then every four or six hours till the stools have resumed their natural color. Tannigen is both astringent and antiseptic, and will not only check the diarrhea, but will also destroy the fermentative changes taking place in the bowels. I do not claim that it will cure every case, but I do claim that, if properly used, it will cure much more promptly than any other medicine.

If there is great pain, I add a little deodorized tincture of opium; but all opiates should be avoided if possible. It is better to allow the patient to suffer some pain than to be stupefied by opiates.

In connection with the foregoing medicines, I wash out the bowels thoroughly two or three times daily with Marchand's hydrozone, 1 to 32 or 40, or hydrogen peroxide, 1 to 16 or 20. I use a fountain syringe with a soft rubber catheter, No. 23 or 24, or even larger may be used. Hold the syringe just high enough above the patient to allow a gentle flow. If the fever is high, I use the water barely tepid; sometimes cool water is better, as it reduces the temperature more rapidly. For general use I have the water pleasantly warm.

In irrigating the bowels, do not be afraid to use plenty of water. Use it freely, not only till the bowels move, but till the water returns clear, showing that the bowels are well cleansed. The water removes all fecal matter and unhealthy mucus, and the hydrozone or peroxide of hydrogen destroys all sepsis and stimulates the intestine to a rapid return to its normal condition.

When there is constant griping and much tympanites, I use the irrigation oftener, every two or three hours if necessary, to keep the patient comfortable.

When nausea and vomiting becomes a marked symptom, leave off all medicine and allow no milk diet at all. Do not return to the milk diet until all vomiting has subsided.

Lime-water has been extolled by all as the great antacid, the sovereign remedy to put with the milk to render it more readily retained and more easily digested. If you will consider how little lime is actually contained in the water, you will at once see that it is the dilution of the milk, and not the lime, that does good. Barley-water is far preferable.

In all cases of more than a week's duration, stimulation is indicated. For this purpose nothing equals alcohol—diluted, of course. Never give alcohol or whisky undiluted, because of the irritant effects upon the mucous membrane of the stomach. There is no rule as to quantity; each case must be a rule unto itself. Give it for its effects, not ill effects from over-stimulation, but enough to support the vital powers. Do not wait until the patient is beginning to show exhaustion before giving it, but give it to prevent exhaustion. In those cases where there is marked emaciation and a tendency to spurious hydrocephalus, active stimulation is the only hope. If that fails to relieve, death must follow.

Thus with tannigen, the ideal intestinal astringent; bismuth, the reliable antacid; water, the great cleanser, and hydrozone, the most potent antiseptic, with strict attention to diet and clothing, we may reasonably expect a majority of our cases to recover.

